# Diagnostic and prognostic value of circulating microRNA-21 in heart failure: A systematic review and meta-analysis

**DOI:** 10.17305/bb.2025.13164

**Published:** 2025-12-24

**Authors:** Annisa Salsabilla Dwi Nugrahani, Wynne Widiarti, Roy Novri Ramadhan, Citrawati Dyah Kencono Wungu, Hendri Susilo, Indah Mohd Amin

**Affiliations:** 1 Master of Medical Science in Clinical Investigation, Harvard Medical School, Boston, MA, USA; 2 Medical Program, Faculty of Medicine, Universitas Airlangga (Airlangga University), Surabaya, Indonesia; 3 Department of Physiology and Medical Biochemistry, Faculty of Medicine, Universitas Airlangga (Airlangga University), Surabaya, Indonesia; 4 Department of Cardiology and Vascular Medicine, Faculty of Medicine, Universitas Airlangga (Airlangga University), Dr. Soetomo General Hospital, Surabaya, Indonesia; 5 Centre of Preclinical Science, Faculty of Dentistry, Universiti Teknologi MARA (UiTM), Sungai Buloh, Selangor, Malaysia

**Keywords:** Circulating miRNA, micro-RNA, miRNA-21, heart failure, diagnostic value

## Abstract

Heart failure (HF) remains a leading cause of global mortality, underscoring the urgent need for reliable, minimally invasive biomarkers to facilitate early diagnosis and risk stratification. MicroRNA-21 (miR-21) has been implicated in cardiac fibrosis, hypertrophy, and the progression of HF; however, its clinical utility remains uncertain. This study presents a systematic review and diagnostic test accuracy (DTA) meta-analysis aimed at assessing the diagnostic and prognostic performance of circulating miR-21 in HF. We estimated pooled sensitivity, specificity, and area under the curve (AUC) for the DTA analysis, and synthesized hazard ratios (HRs) with 95% confidence intervals (CIs) for prognostic outcomes. Additionally, univariate meta-regression was conducted to explore demographic and clinical moderators. Our analysis included fourteen studies with a total of 1327 participants. Results demonstrated that circulating miR-21 levels were significantly elevated in HF patients compared to controls (fold change 1.61; 95% CI 1.46–1.78; *P* < 0.001). The diagnostic accuracy was notably high, with a sensitivity of 0.94 (95% CI 82.0–98.0), specificity of 0.90 (95% CI 79.0–96.0), and AUC of 0.97 (95% CI 96.0–98.0). Elevated levels of miR-21 were associated with an increased risk of worsening HF severity (HR 1.84; 95% CI 1.14–2.97; *P* ═ 0.01) and HF-related cardiovascular death (HR 2.00; 95% CI 1.30–3.03; *P* ═ 0.001). However, no significant association was found with HF-related hospitalization (HR 0.97; 95% CI 0.61–1.52; *P* ═ 0.88). Variability in sample type and differing clinical thresholds contributed to heterogeneity across studies. These findings support the potential of circulating miR-21 as a diagnostic and prognostic biomarker for HF. Nevertheless, further research with standardized sample sizes and clinical thresholds is necessary to establish robust evidence for its clinical application.

## Introduction

Heart failure (HF) is a complex global health challenge affecting over 56 million individuals worldwide [1]. As the leading cause of morbidity and mortality, the 5-year prognosis for HF remains poor, with survival rates below 45% [2]. Although HF often arises from myocardial infarction (MI), current clinical risk scoring systems and biomarkers do not facilitate early detection of HF post-MI. Factors such as ventricular function, aging, obesity, renal failure, and atrial arrhythmias can complicate the clinical interpretation of natriuretic peptides (NP) [3]. Timely and accurate detection of HF can enable prompt interventions to improve patient outcomes, highlighting an urgent need for reliable, minimally invasive biomarkers for diagnosing and assessing HF. Consequently, innovative strategies must be developed to enhance risk stratification and prevent long-term health complications [4].

MicroRNAs (miRNAs) are non-coding, single-stranded RNAs approximately 22 nucleotides in length that modulate gene expression post-transcriptionally by inhibiting translation or promoting the degradation of target mRNAs [5]. Among the identified miRNAs, miRNA-21 is one of the most extensively studied in cardiovascular (CV) diseases. It has been associated with critical pathological pathways in the development of HF, including cardiac remodeling, apoptosis, and hypoxic signaling [6]. Elevated levels of miRNA-21 have been linked to an increased risk of major adverse CV events (MACE) in diabetic patients [7] and have been found significantly elevated in elderly patients with non-ST elevated MI (NSTEMI), indicating a role in cardiac fibrosis [7]. Notably, miRNA-21 has been reported to differentiate between HF with reduced ejection fraction (HFrEF) and HF with preserved ejection fraction (HFpEF), correlating with echocardiographic parameters [8], thus suggesting its potential as a biomarker for HF.

In the context of HF, circulating miRNA-21 predominantly originates from cardiomyocytes, cardiac fibroblasts, and vascular endothelial cells subjected to mechanical stress, ischemia, or inflammatory stimuli [9]. Under these pathological conditions, miRNA-21 is actively secreted into the bloodstream via exosomes and microvesicles or bound to RNA-binding proteins such as Argonaute-2, which protect it from enzymatic degradation. Increased circulating levels of microRNA-21 (miRNA-21) reflect its intracellular upregulation in the failing myocardium, where it regulates fibroblast activation, extracellular matrix remodeling, and cardiomyocyte apoptosis via pathways such as phosphatase and tensin homolog (PTEN)/protein kinase B (AKT) and transforming growth factor-beta (TGF-β)/SMAD signaling. The presence of miRNA-21 in circulation serves as both a consequence of myocardial stress and a potential intercellular signaling mechanism, linking myocardial pathology with measurable molecular changes in plasma or serum [10, 11].

Despite existing evidence, the clinical utility of circulating miRNA-21 in diagnosing and predicting the prognosis of HF remains uncertain. Therefore, this study aims to evaluate the diagnostic accuracy and prognostic value of miRNA-21 concerning HF incidence, hospitalization, and mortality through a systematic review and meta-analysis, including meta-regression to explore factors influencing its performance.

## Material and methods

This systematic review and meta-analysis adhered to the Preferred Reporting Items for Systematic Reviews and Meta-Analysis (PRISMA) guidelines [12] and was registered with International Prospective Register of Systematic Reviews (PROSPERO) under the number CRD42024576851 on August 24, 2024.

### Search strategy and data sources

A comprehensive search strategy was devised to gather relevant articles from MEDLINE, Web of Science, EMBASE, Scopus, and gray literature up to August 2024. Manual searches of references were conducted to ensure thorough inclusion. The search was independently performed by three authors (ASDN, WW, RNR) using the query formulation “microRNA-21” OR “miRNA-21” AND “heart failure,” along with their synonyms, detailed using Boolean operators. Searches were restricted to English language studies involving human subjects. The full search strategy used in each database is available in Supplementary Material 1.

### Study selection and eligibility criteria

Studies included in this meta-analysis must: a) involve individuals diagnosed with HF based on clinical manifestations or echocardiography according to established guidelines, b) measure miRNA-21 levels during an HF incident using standardized methods such as qPCR, miRNA sequencing, or real-time PCR, c) utilize serum or plasma samples for circulating miRNA-21, and d) consist of observational, prospective, or retrospective studies, including diagnostic test accuracy (DTA) studies. Studies providing diagnostic values such as sensitivity, specificity, or area under the curve (AUC), along with their 95% confidence intervals (CIs), or correlation values between miRNA-21 and HF were included in the diagnostic meta-analysis. Studies included in the prognostic meta-analysis must provide data on prognostic endpoints, such as New York Heart Association (NYHA) functional class, hospitalization, and HF-related mortality.

Studies lacking sufficient data for analysis, duplicate studies, reviews, case reports, or those involving non-human subjects were excluded. Study selection and screening were independently performed by three authors (ASDN, WW, RNR), with any disagreements resolved through discussion with a referee (HS, CDK).

### Data extraction

The following details were extracted from each study: author, publication year, study design, country, sample size, diagnostic values (specificity, sensitivity, AUC), predictive values (HF incidence, hospitalization, NYHA class, CV deaths), and cut-off values. The extracted information was compiled in Microsoft Excel 2021. The comprehensive search and data extraction processes were conducted independently by three authors (ASDN, WW, RNR) to assess study eligibility, with any discrepancies resolved through discussion.

### Quality assessment

The risk of bias in diagnostic test studies was assessed using the Quality Assessment of Diagnostic Accuracy Studies 2 Revised (QUADAS-2) tool, while the Quality in Prognosis Studies (QUIPS) tool was employed for prognostic studies [13]. The QUADAS-2 tool evaluated four phases in DTA studies, including patient selection, index test, reference standard, and flow with timing. Conversely, the QUIPS tool assessed six domains: study participation, attrition, prognostic factor measurement, outcome measurement, study confounding, and statistical analysis with reporting. The QUIPS tool categorizes the risk of bias as low (green), moderate (yellow), or high (red).

### Statistical analysis

To synthesize the results, several analyses were performed in this study. First, a bivariate random-effects model meta-analysis for DTA studies was conducted to pool the outcomes of sensitivity, specificity, and AUC, along with their 95% CIs from each study. When sensitivity data were unavailable, it was calculated as the proportion of true positives (TP) out of the total number of individuals with the disease (TP plus false negatives [FN]). Conversely, specificity was calculated as the proportion of true negatives (TN) out of the total number of individuals without the condition (TN plus false positives [FP]). Summary receiver operating characteristic (SROC) models and plots were utilized to visualize the pooled sensitivity and specificity data. For studies providing different thresholds for individual analyses, the threshold effect was quantified by performing a Spearman correlation analysis between the logit of sensitivity and the logit of (1-specificity).

Meta-analyses of prognostic studies pooled hazard ratios (HRs) with 95% CIs for three endpoints: (1) NYHA class progression, (2) HF-related hospitalization, and (3) HF-related CV death. Prespecified subgroup analyses explored heterogeneity based on acute vs chronic HF, baseline severity (stage/NYHA), and the quantification methods used. Between-study heterogeneity was assessed using Cochran’s *Q* and *I*^2^ statistics. A coherent decision rule was applied: fixed-effect inverse-variance models were used when *I*^2^ < 50% and between-study variance was negligible, while random-effects models (restricted maximum likelihood with Hartung–Knapp adjustment) were applied when *I*^2^ ≥ 50% or τ^2^ was non-zero. Sensitivity analyses (leave-one-out and exclusion of abstracts) tested the robustness of the findings. Small-study effects were examined using Egger’s test, and when indicated, bias-adjusted estimates were derived using trim-and-fill [14]. All analyses were conducted in Stata BE version 19 (College Station, TX, USA).

### Meta-regression and sensitivity analysis

If study heterogeneity was considered moderate (50%–75%) or high (>75%), a univariate random-effects meta-regression analysis was conducted to assess the sources of heterogeneity. Sensitivity analysis was performed using the “leave-one-out” method to ensure the robustness of findings by omitting one study at a time.

### Publication bias

Two different methods for assessing publication bias in this meta-analysis were utilized. Publication bias for diagnostic test meta-analysis was measured using Deeks’ funnel plot [15]. A statistically significant asymmetry value, indicated by a slope coefficient with a *P* value of less than 0.10, was considered indicative of publication bias. Additionally, Egger’s test was employed to identify publication bias or small-study effects for prognostic meta-analysis, with a *P* value of less than 0.05 considered significant.

## Results

### Study selection

**Figure 1.**
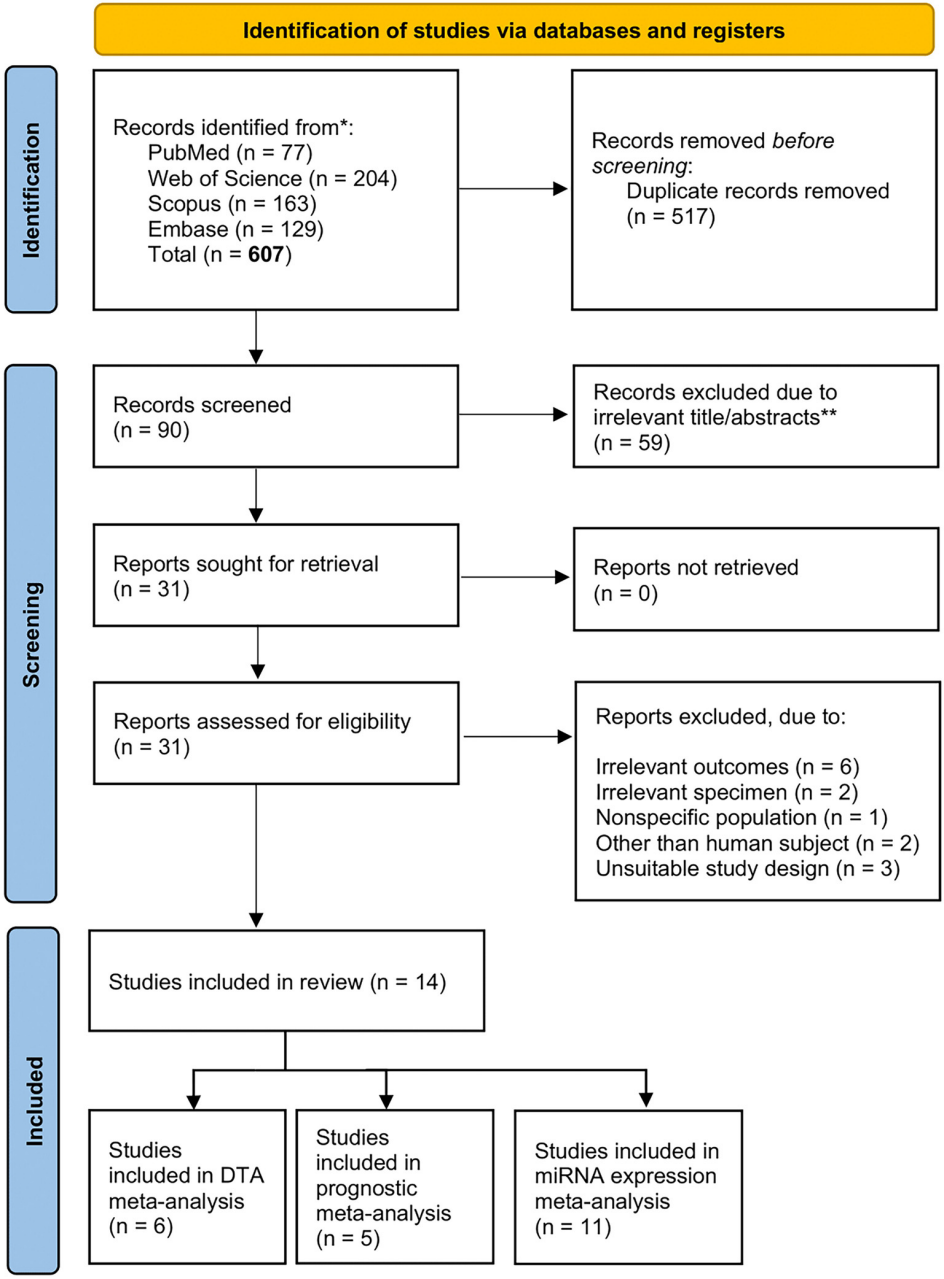
**PRISMA flow diagram of included studies**.

A total of 607 study records were identified from four scientific databases. After removing 517 duplicate records, 90 studies were screened based on title and abstract. Following this screening, 59 articles were excluded due to irrelevance to the topic. The remaining 31 records were pursued for retrieval. During the eligibility assessment, 17 studies were excluded due to irrelevant outcomes (*n* ═ 8), irrelevant specimens (*n* ═ 3), non-specific populations (*n* ═ 2), the use of non-human subjects (*n* ═ 2), and unsuitable study designs (*n* ═ 2). Ultimately, 14 studies were included in this meta-analysis. Of these, six studies and seven datasets were analyzed for diagnostic accuracy, five for prognostic value, and 11 for expression patterns, with certain studies contributing to multiple analyses due to overlapping outcomes. Details of the study selection process are presented in Figure 1.

### Characteristics of the included studies

Fourteen studies were ultimately included in this analysis [4, 16–28]. The studies were conducted in Asia, Europe, and America and encompassed various types of HF, including acute decompensated HF (ADHF) and chronic HF (CHF), with both systolic and diastolic dysfunctions. Studies measuring circulating miRNA-21 from both plasma and serum were included. Measurement methods for miRNA-21 predominantly involved RT-PCR, RT-qPCR, microarray analyses, and next-generation sequencing. Several studies reported specific cut-off values for biomarkers, often utilizing the Youden Index to determine sensitivity and specificity, with some demonstrating high diagnostic accuracy. Characteristics of the included studies are summarized in Table 1.

**Table 1:** Characteristics of the included studies

**Study, year**	**Country**	**Study design**	**Acute/chronic HF**	**LVEF**	**No. patients with HF**	**No. control**	**Assay methods**
Al-Hayali et al., 2019 [16]	Turkey	Case-control	ADHF	HFrEF	45	45	RT-PCR
Ben-Zvi et al., 2020 [17]	Israel	Case-control	ADHF	HFrEF	39	21	RT-qPCR
Cakmak et al., 2015 [18]	Turkey	Prospective cohort	CHF	HFrEF	42	15	Microarray
Davydova et al., 2020 † [19]	Russia	Prospective cohort	CHF	HFpEF	180	60	RT-PCR
Ding et al., 2020 [28]	China	Case-control	All HF	all	62	62	RT-PCR
Galluzzo et al., 2021 [20]	Italy	Case-control	CHF	HFrEF	30	36	RT-PCR
Goren et al., 2012 [21]	Israel	Case-control	CHF	HFrEF	30	30	RT-qPCR
Kan et al., 2019 [22]	China	Case-control	CHF	HFpEF	60	35	RT-PCR
Marketou et al., 2024 [23]	Greece	Prospective cohort	ADHF	HFpEF	56	94	RT-qPCR
Meiri et al., 2020 [24]	Israel	Prospective cohort	CHF	HFrEF	8	10	RT-qPCR
Rincón et al., 2022 [4]	Spain	Prospective cohort	CHF	all	43	268	RT-qPCR
Schneider et al., 2018 [25]	Brazil	Prospective cohort	ADHF	HFrEF	48	17	RT-qPCR
Sygitowicz et al., 2015 [27]	Poland	Prospective cohort	Both	HFrEF	35	26	RT-qPCR
Zhang et al., 2017 [26]	China	Prospective cohort	ADHF	HFrEF	80	40	RT-qPCR

† Abstract only. Abbreviations: HF: Heart failure; LVEF: Left ventricular ejection fraction; ADHF: Acute decompensated heart failure; CHF: Chronic heart failure; HFrEF: Heart failure with reduced ejection fraction; HFpEF: Heart failure with preserved ejection fraction; RT-PCR: Reverse transcription polymerase chain reaction; RT-qPCR: Reverse transcription quantitative polymerase chain reaction.

Diagnostic studies were assessed using the QUADAS-2 while prognostic studies were evaluated using the QUIPS for quality assessment [13]. The quality of the included studies is summarized in Supplementary Material 2.

### Diagnostic values of miRNA-21 in HF

Six DTA studies, comprising seven datasets (*n* ═ 507 participants), were included. One study provided two outcomes from different samples, one from peripheral vein (PV) and the other from coronary sinus (CS) [26]. Pooled estimates from six studies and seven datasets (Zhang 2017 CS and PV reported separately) indicated that circulating miR-21 detected HF with a sensitivity of 0.94 (95% CI 82.0–98.0) and specificity of 0.90 (95% CI 79.0–96.0). Heterogeneity was high for both sensitivity (*I*^2^ = 85.0%) and specificity (*I*^2^ = 82.4%). The summary ROC analysis yielded an AUC of 0.97 (95% CI 0.95–0.97). Deeks’ funnel plot exhibited a non-significant slope (*P* ═ 0.60), suggesting no significant publication bias.

Our study explored three clinically plausible scenarios using Fagan’s nomogram to illustrate a range of pre-test probability settings. For a pre-test probability of 25%, the application of Fagan’s nomogram indicated that a positive test result would raise the post-test probability to 76%, while a negative result would decrease it to 3%. In scenarios where the initial pre-test probability was set at 10%, a positive test increased the post-test probability to 51%, and a negative result would decrease it to 1%. Furthermore, when the pre-test probability was 50%, a positive result elevated the post-test probability to 90%, while a negative result would decrease it to 6%. These findings collectively underscore the potential utility of miRNA-21 in supporting the diagnosis of HF across various clinical contexts. Further details regarding the selection and rationale for these pre-test probabilities are provided in Supplementary Material 3.

**Figure 2.**
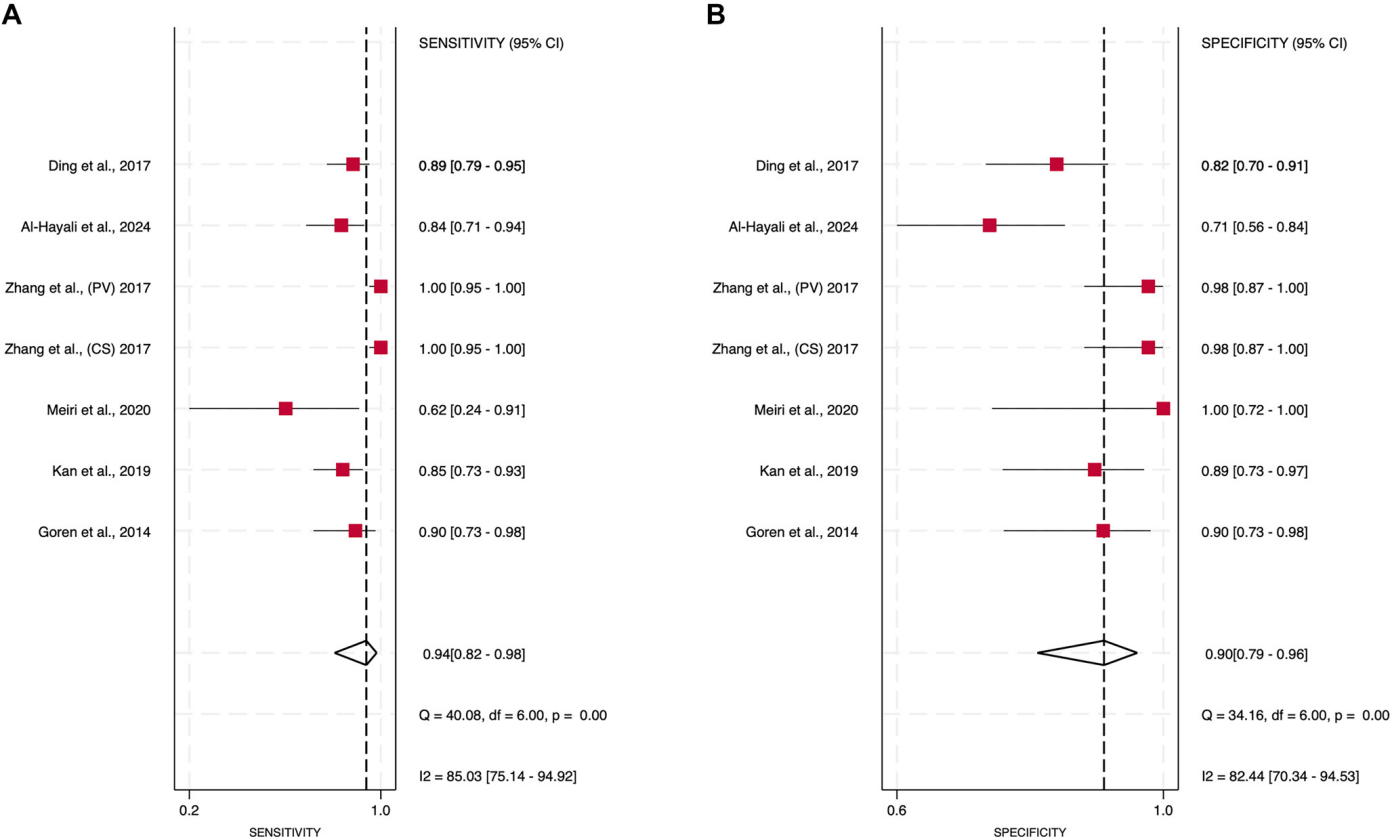
**Forest plots summarizing the diagnostic accuracy of circulating miRNA-21 for heart failure (HF): (A) sensitivity and (B) specificity.** Point estimates from six studies (seven independent datasets; Zhang et al., 2017 reported CS and PV cohorts separately) are represented as red squares with 95% confidence intervals (horizontal bars). The pooled summary estimate is illustrated by the diamond (width = 95% CI), while the dashed vertical line indicates the pooled value. Measures of between-study heterogeneity, including Cochran’s *Q* and *I*^2^, are provided below each panel.

**Figure 3.**
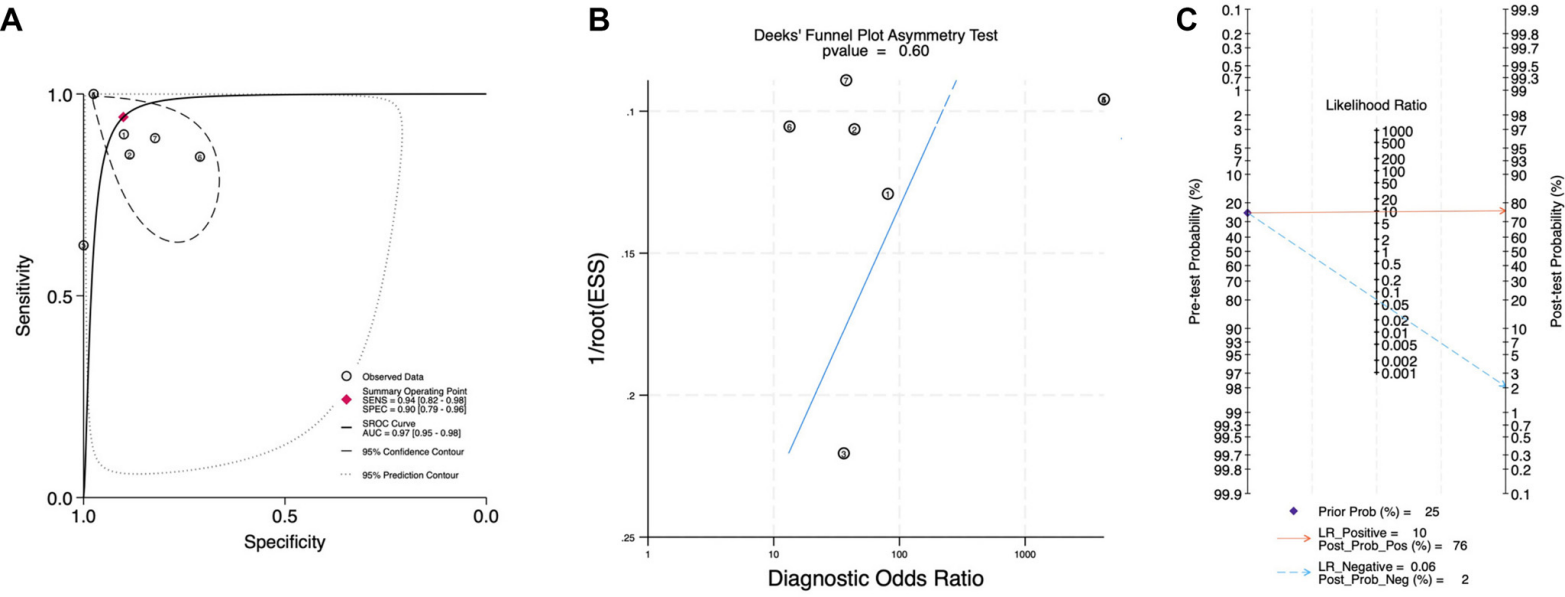
**Diagnostic accuracy and clinical utility of circulating miRNA-21 for heart failure (HF).** (A) SROC plot showing individual study estimates (circles), the bivariate summary point (diamond), fitted SROC curve, and 95% confidence/prediction regions; (B) Deeks’ funnel plot for publication bias (*P* ═ 0.60); (C) Fagan’s nomogram translating likelihood ratios to post-test probability (pre-test 25%: post-test 76% for LR+ ═ 10; 2% for LR-- ═ 0.06).

Details of the forest plot for specificity and sensitivity results are available in Figures 2 and 3. Supplementary Material 4 provides the total counts of TP/FP/TN/FN for each study. However, a significant threshold effect was noted, with Spearman’s rho ═ −0.78 and a *P* value of 0.045 detected in our analysis, suggesting that heterogeneity in our meta-analysis is at least partly due to differences in test positivity thresholds across studies.

### Upregulated miRNA-21 levels in HF patients

**Figure 4.**
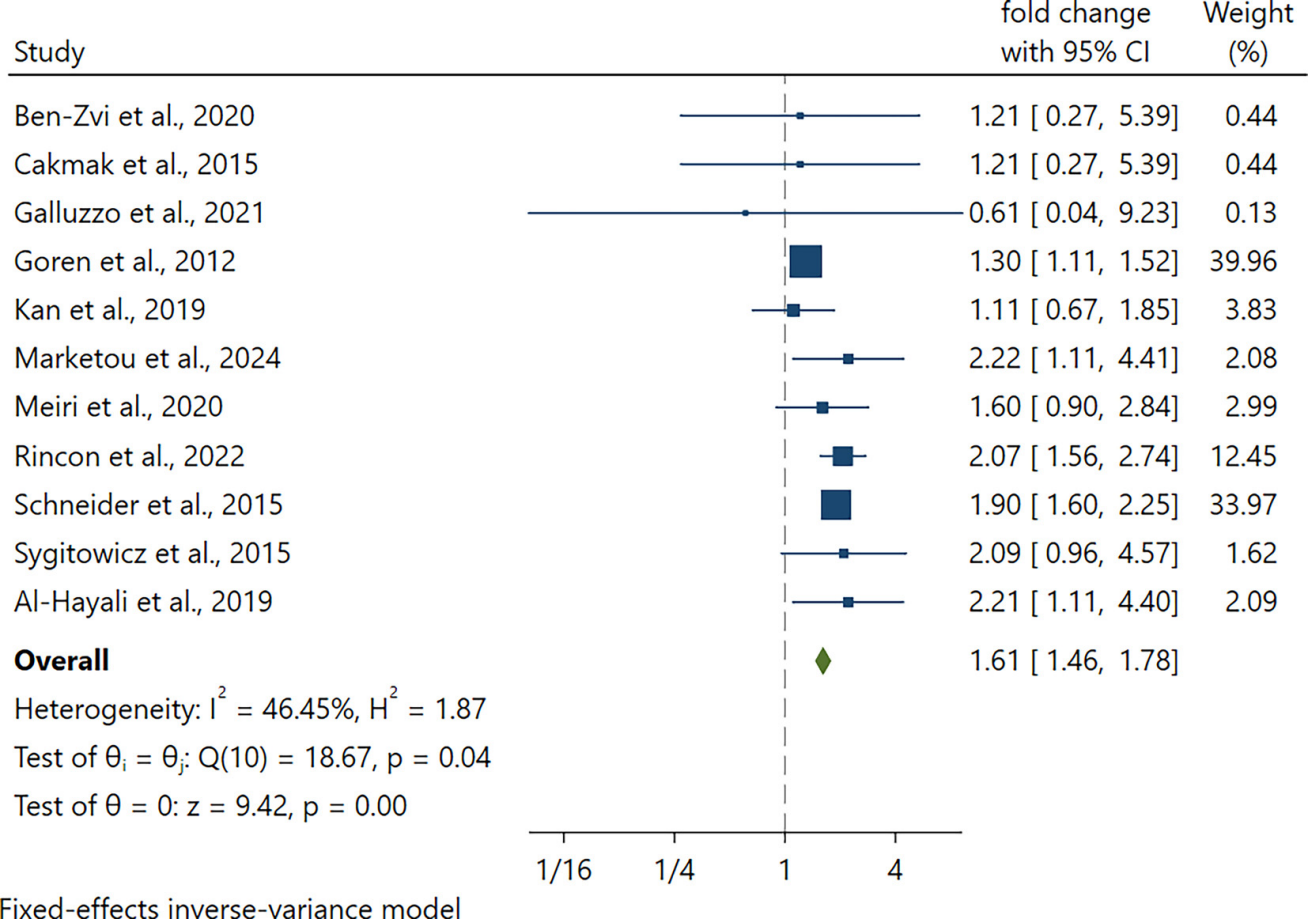
**Forest plot depicting miRNA-21 expression levels in relation to HF events.** Individual squares represent study-specific fold changes in circulating miR-21 expression among HF patients compared to non-HF controls. Horizontal lines indicate 95% confidence intervals, with the size of each square proportional to the inverse-variance weight of the respective study. The diamond shape represents the pooled effect estimate derived from a fixed-effects inverse-variance model, illustrating significantly elevated circulating miR-21 levels in HF patients (overall fold change 1.61; 95% CI 1.46–1.78; *P* < 0.001). The analysis reveals low-to-moderate heterogeneity between studies, with an *I*^2^ value of approximately 46%. Abbreviations: HF: Heart failure; miRNA-21: MicroRNA-21; CI: Confidence interval.

We conducted a pooled analysis of 11 studies evaluating differentially expressed miRNA-21 levels in HF subjects compared to non-HF. The pooled analysis demonstrated that circulating miR-21 was significantly upregulated in HF compared with controls (fold change 1.61; 95% CI 1.46–1.78; *P* < 0.001) (Figure 4). Between-study heterogeneity was low (*I*^2^ = 47.2%), thus a fixed-effect model was employed. To explore potential sources of heterogeneity, meta-regression was performed, which will be detailed in the next section (Table 2).

**Table 2:** Meta-regression analysis from the pooled studies

**Category**	**Variable**	**Coefficient**	**Std. Err.**	**z**	***P*>|z|**	**95% Conf. Interval (Lower)**	**95% Conf. Interval (Upper)**
Onset	ADHF	0.32	0.46	0.71	0.48	--0.57	1.22
	CHF	--0.01	0.42	--0.02	0.98	--0.84	0.82
LVEF	HFpEF	0.72	1.56	0.46	0.65	--2.35	3.79
	HFrEF	--0.20	0.52	--0.38	0.71	--1.22	0.83
Specimen	Plasma	Ref.					
	serum	0.36	0.39	0.93	0.35	--0.40	1.13
Quantification method	RT-qPCR	Ref.					
	RT-PCR	0.66	1.58	0.42	0.68	--2.43	3.75
	microarray	0.59	1.75	0.34	0.74	--2.84	4.02
Continent	America	Ref.					
	Asia	--0.41	0.85	--0.48	0.63	--2.08	1.26
	Europe	--0.14	1.28	--0.11	0.91	--2.65	2.37
Constant	cons	--0.15	2.04	--0.07	0.94	--4.15	3.85

Abbreviations: ADHF: Acute decompensated heart failure; CHF: Chronic heart failure; LVEF: Left ventricular ejection fraction; HFpEF: Heart failure with preserved ejection fraction; HFrEF: Heart failure with reduced ejection fraction; RT-qPCR: Reverse transcription quantitative polymerase chain reaction; RT-PCR: Reverse transcription polymerase chain reaction.

### Subgroup analysis

**Figure 5.**
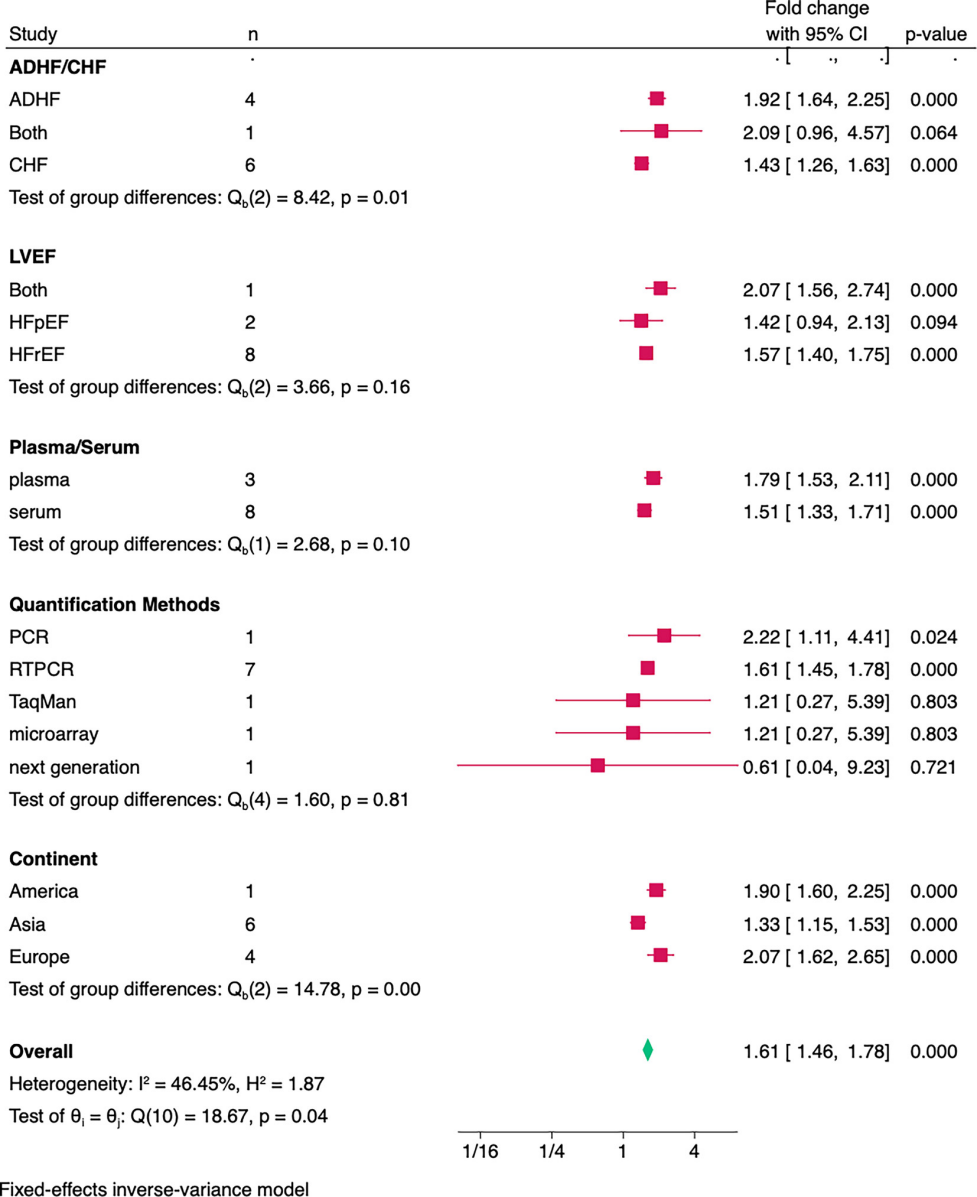
**Subgroup analysis of circulating miRNA-21 expression levels in HF.** Forest plot shows fold changes (squares) with 95% confidence intervals across subgroups defined by onset (ADHF, CHF, both), LVEF phenotype (HFrEF, HFpEF, both), sample type (plasma vs serum), quantification method, and continent; the diamond indicates the overall pooled fold change from a fixed-effects inverse-variance model. Abbreviations: HF: Heart failure; ADHF: Acute decompensated heart failure; CHF: Chronic heart failure; HFrEF: Heart failure with reduced ejection fraction; HFpEF: Heart failure with preserved ejection fraction; LVEF: Left ventricular ejection fraction.

Subgroup analyses were conducted based on clinical onset (acute or chronic HF), left ventricular ejection fraction (LVEF) category (reduced vs. preserved), sample type, quantification method, and study continent. The pooled effect size significantly differed across onset and continents. Specifically, the pooled effect size varied significantly by clinical onset (*P* ═ 0.01), with studies including only ADHF patients showing a 1.92-fold increase, those including only CHF patients showing a 1.43-fold increase, and studies including both ADHF and CHF patients demonstrating a 2.09-fold increase. Effect sizes also varied significantly by continent (*P* < 0.01), with studies conducted in Europe showing a 2.07-fold increase, those in Asia a 1.33-fold increase, and those in America a 1.90-fold increase. No other interaction tests reached statistical significance (*P >* 0.05). Details of subgroup analysis is available in Figure 5.

### Association between miRNA-21 and NYHA class

**Figure 6.**
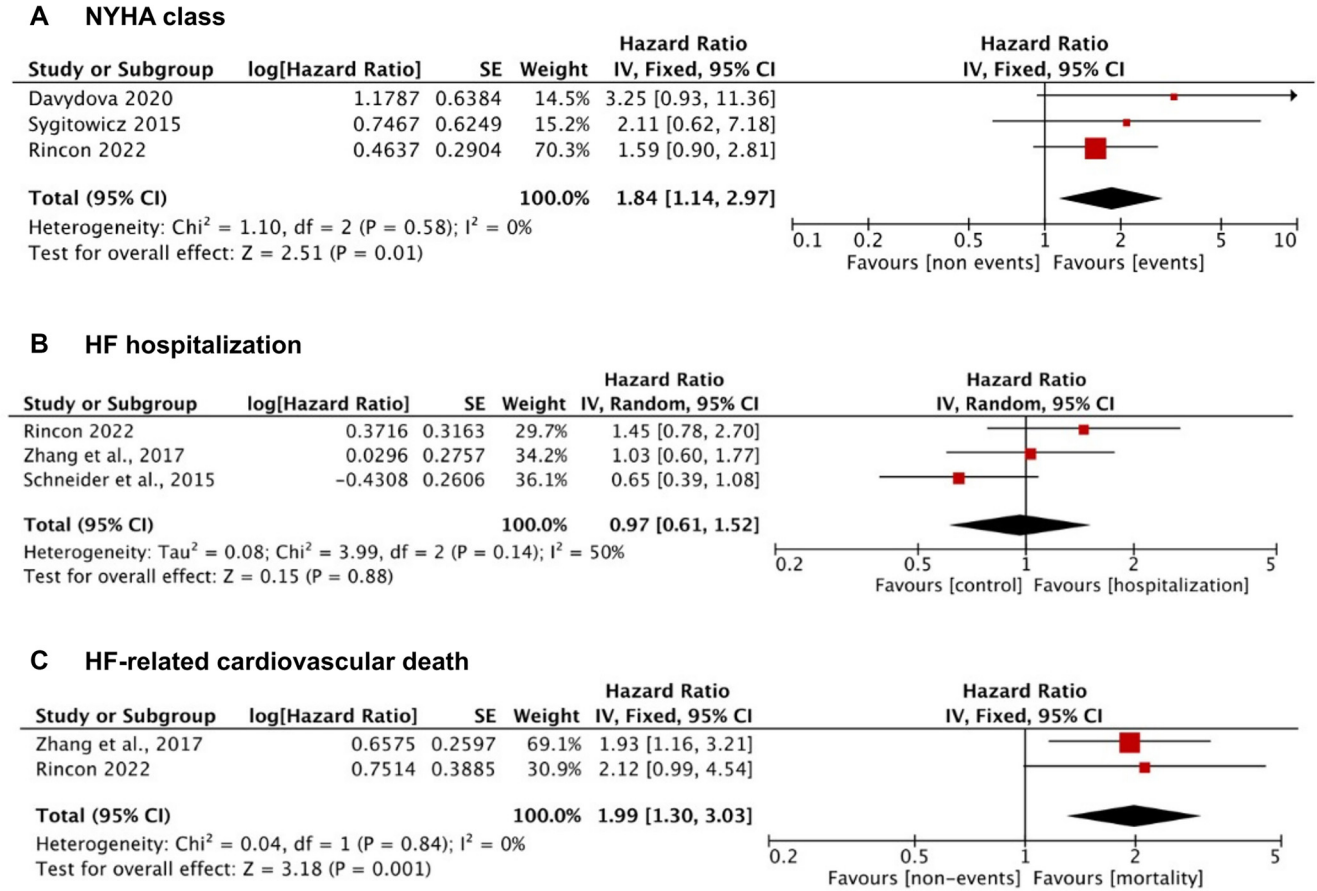
(A) Forest plot illustrating the relationship between miRNA-21 and NYHA classification. (B) Forest plot evaluating the association between miRNA-21 and heart failure-related hospitalizations. (C) Forest plot examining the relationship between miRNA-21 and heart failure-related cardiovascular mortality.

The relationship between elevated miRNA-21 levels and the severity of HF was evaluated through its correlation with the NYHA classification. A fixed-effects meta-analysis of three studies revealed that patients with higher miRNA-21 levels had an 84% increased risk of being classified as NYHA class ≥ 2 compared to those with lower miRNA-21 levels (HR: 1.84, 95% CI: 1.14–2.97, *P* ═ 0.01) (Figure 6A). Low heterogeneity was observed (*I*^2^ ═ 0%), thus justifying the use of a fixed-effects model.

### Prognostic value of miRNA-21 in HF hospitalization

Three studies investigated the association between miR-21 levels and HF-related hospitalization (Figure 6B). The random-effects meta-analysis indicated no significant association (HR: 0.97; 95% CI: 0.61–1.52; *P* ═ 0.88). Moderate heterogeneity was noted (*I*^2^ = 50%), which warranted the application of a random-effects model. Sensitivity analyses confirmed that no single study significantly impacted the pooled effect.

### Prognostic value of miRNA-21 in HF-related CV death

Two studies examined the relationship between miR-21 levels and HF-related CV death. The pooled estimate suggested that higher miR-21 levels were associated with a two-fold increased risk (HR: 2.00; 95% CI: 1.30–3.03; *P* ═ 0.001) (Figure 6C). The heterogeneity was negligible (*I*^2^ = 0%), with both studies consistently demonstrating a positive association, thus supporting the fixed-effects model.

### Meta-regression analysis

Meta-regression was conducted to identify potential sources of heterogeneity in the pooled estimates from expression and diagnostic studies. This analysis assessed variables such as study location by continent, sample type, HF subtype HFrEF vs HFpEF, miRNA quantification method, and the status of the disease (acute vs chronic).

None of these variables reached statistical significance in expression studies, indicating that they did not substantially explain the heterogeneity observed among studies. Differences in sample type (plasma vs serum), HF subtype, or quantification methods (including RT-qPCR, RT-PCR, microarray, and next-generation sequencing) were not associated with significant alterations in effect estimates.

In contrast, meta-regression analysis for diagnostic studies revealed a significant influence of sample type (plasma/serum) on sensitivity (*P* < 0.01) and specificity (*P* ═ 0.02). Detailed statistical analyses for meta-regression are presented in Table 2.

### Publication bias

Publication bias was evaluated using Deeks’ funnel plot for the DTA meta-analysis and Egger’s test ± trim-and-fill for the expression studies meta-analysis. The DTA meta-analysis revealed no evidence of asymmetry in Deeks’ funnel plot (*P* ═ 0.60). For the expression studies meta-analysis, no significant publication bias or small-study effects were detected, as indicated by Egger’s test (*P* ═ 0.80) and visual inspection of the trim-and-fill funnel plot (Supplementary Material 5). The bias estimate (intercept = 0.11, SE = 0.46) was not statistically significant, reinforcing the absence of small-study effects.

### Sensitivity analysis

Sensitivity analysis was performed to assess the robustness of the meta-analysis results by systematically excluding individual studies. This method aimed to determine whether any single study or combination of studies significantly influenced the overall findings. As shown in Supplementary Material 3, the exclusion of any single study had no substantial impact on the overall results, indicating that the conclusions remain robust even when individual studies are omitted.

## Discussion

MiRNA-21 demonstrated a pooled sensitivity of 94% and a specificity of 90% in the diagnosis of HF, yielding a SROC–AUC of 0.97. Fagan’s nomogram further confirms its potential clinical utility, suggesting that the positive post-test probability of diagnosing HF significantly increases. Prognostically, the upregulation of miRNA-21 is significantly associated with HF severity and outcomes, with higher levels correlating with an 84% increased risk of being classified into a higher NYHA class. This finding was consistent across studies with low heterogeneity, underscoring miRNA-21’s role as a biomarker for disease severity. Additionally, elevated miRNA-21 levels were predictive of HF-related CV death, indicating a two-fold increase in risk. However, no significant correlation was found between miRNA-21 levels and HF-related hospitalization, potentially reflecting limitations in the available data or variability in hospitalization criteria across studies.

Initially investigated in the context of tumor growth, miRNA-21 is now recognized for its role in maintaining CV homeostasis. In HF, it is known to promote cardiac fibrosis by activating the extracellular signal-regulated kinase–mitogen-activated protein kinase (ERK-MAPK) pathway through the suppression of sprout homolog 1 (SPRY1) and modulating Ang II-induced cardiac fibrosis via inhibition of PTEN and SMAD family member 7 (SMAD7) [29]. The other strand of miRNA-21, miRNA-21-3p, has also been implicated in the progression of cardiac hypertrophy to HF, with increased expression noted during HF in humans inducing cardiomyocyte hypertrophy through exosome communication between cardiac fibroblasts and cardiomyocytes [30].

In summary, miRNA-21 is upregulated during the transition from cardiac hypertrophy to HF, promoting cardiomyocyte hypertrophy and mediating cardiac fibrosis, thus highlighting its significant role in HF pathogenesis [28]. This is further supported by Pan et al., who linked miRNA-21 to critical remodeling and fibrosis processes by regulating connective tissue growth factor (CTGF). It is known to be activated by hypoxia and inflammation, influencing cardiomyocyte survival [31]. Understanding the precise, cell-specific functions and targets of miRNA-21 is vital for its potential clinical application in treating CV diseases.

According to pooled outcomes, changes in miRNA-21 levels are closely associated with HF, particularly in predicting its incidence, NYHA functional class progression, hospitalization rates, and CV mortality. Consistent with previous studies, circulating miRNA-21 levels in patients with stable or ADHF differ significantly from those in control subjects, supporting their use as biomarkers in HF diagnosis. However, the role of miRNA-21 varies between acute and chronic HF. In acute phases, miRNA-21 is typically upregulated, contributing to the immediate response to cardiac injury by promoting tissue repair and reducing infarct size. As the disease progresses to a chronic state, the expression of miRNA-21 generally returns to baseline, indicating a stabilization phase where acute repair mechanisms are less active [32].

A study by Ding et al. [28] reported an AUC of 0.944 for miRNA-21 in diagnosing HF, with a sensitivity of 89.7% and specificity of 82.8%. This high diagnostic accuracy is attributed to the stability of miRNA-21 in blood, allowing it to regulate sequence-specific gene expression without being influenced by hemolysis, age, or gender in HF diagnosis. StudiesResearch indicates that miRNA-21 modulates critical signaling pathways, such as ERK-MAPK, which are essential in developing cardiac hypertrophy and fibrosis. Its ability to influence key processes in HF pathophysiology supports its use as a diagnostic tool for identifying patients at risk for HF and guiding therapeutic interventions [33, 34]. Previous studies have shown that miRNA-21 levels correlate significantly with the severity of HF, offering a non-invasive method for early HF detection and monitoring disease progression [26].

Furthermore, circulating miRNA levels correlate with established prognostic clinical parameters, such as Brain NP (BNP), N-terminal prohormone BNP (NT-proBNP), and LVEF [27]. This reinforces the potential of miRNA-21 as a biomarker for HF progression and highlights the complexity of its role in various aspects of HF pathophysiology. Ongoing research underscores the need for further studies to fully understand the multifaceted roles of miRNA-21 and its implications for HF management [32]. BNP and its inactive fragment, NT-proBNP, remain the current gold standard biomarkers for diagnosing and monitoring HF, reflecting myocardial wall stress and hemodynamic overload. However, these peptides can be influenced by factors such as age, renal function, obesity, and atrial fibrillation, which may limit their diagnostic accuracy in specific patient populations [35]. In contrast, miRNA-21 reflects molecular and structural remodeling processes rather than hemodynamic changes, directly participating in the regulation of fibrosis, inflammation, and cardiomyocyte survival. Several studies have shown that circulating miRNA-21 levels correlate with BNP and NT-proBNP but may also detect subclinical myocardial remodeling even when NP levels are normal. A recent study demonstrated that miRNA-21-5p levels were significantly elevated in patients with HFrEF and correlated moderately with NT-proBNP, suggesting that miRNA-21 may complement, rather than replace, traditional biomarkers in comprehensive HF assessments. The pooled results of included studies also indicated that the diagnostic accuracy of miRNA-21 is notably higher in diabetic patients. Hyperglycemia, insulin resistance, and associated oxidative stress and inflammation in diabetes enhance the expression and effects of miRNA-21. Analysis of the GSE4745 microarray dataset found that high glucose conditions cause down-regulation of hexokinase 2 (Hk2) miRNA and increased expression of regulatory miRNAs, including miRNA-21, in rats, indicating its significant role in disease development [36]. Therefore, the presence of diabetes, combined with upregulated miRNA-21, can worsen HF outcomes in diabetic patients [37].

Despite advancements, the rising incidence of HF underscores the need for effective diagnostic and treatment options, such as blood biomarkers. While many biomarkers have been identified, most lack the required sensitivity and specificity to reliably detect the progression of HF severity. Thus, miRNA-21 shows promise, as pooled results from included studies demonstrate good sensitivity and specificity [38]. The ability of miRNA-21 to influence critical processes in HF pathophysiology supports its use as a diagnostic tool capable of identifying patients at risk for HF and guiding therapeutic interventions. Furthermore, miRNA-21 is involved in inflammation and immune-related pathways, suggesting its role in regulating inflammation by modulating genes critical to inflammatory responses, a key factor in initiating myocardial remodeling and injury in HF. Its stress-responsive characteristics position miRNA-21 as a pro-fibrotic molecule central to cardiac fibroblast function, particularly enriched in the fibroblasts [39] of HF patients. *In vivo*, inhibition of miRNA-21 prevents pressure overload-induced cardiac interstitial fibrosis and dysfunction. Pathological hypertrophy and fibrosis in myocardial cells lead to increased left ventricular filling pressures and, ultimately, HF syndrome, which is a primary mechanism of HFpEF [38].

In clinical practice, miRNA-21 could serve as a non-invasive biomarker for early detection, prognosis, and monitoring of HF progression. Its integration into routine clinical assays may facilitate patient risk stratification, thereby enabling personalized treatment approaches. Additionally, miRNAs have been reported to differentiate between HFrEF and HFpEF while showing correlations with echocardiographic measurements, potentially addressing the limitations of NT-proBNP [8, 26]. As the field advances, clinical trials will be essential to determine the safety, efficacy, and long-term outcomes of anti-miRNA-21 therapy in humans. However, the cost and accessibility of miRNA-based diagnostics and therapeutics must be considered, especially in resource-limited settings. Moreover, no standardized clinical threshold for circulating miRNA-21 has been established, and variations in assay techniques and normalization methods hinder comparability across studies. Future work should prioritize developing standardized quantification protocols and clinically validated cut-off values to ensure reproducible interpretation and facilitate the integration of miRNA-21 into daily practice. As our understanding of miRNA-21 in HF evolves, its integration into clinical practice may transform the management and outcomes of this complex and prevalent condition.

## Conclusion

Circulating miRNA-21, as a minimally invasive biomarker, demonstrates strong diagnostic and prognostic potential for HF detection and monitoring. Elevated miRNA-21 levels are consistently associated with HF occurrence, NYHA class progression, and CV mortality. These results support its integration as a biomarker for early diagnosis and patient risk stratification, aligning with precision medicine approaches in HF management. However, standardized analytical thresholds and validated cut-off points for miRNA-21 quantification remain to be established. Therefore, large, prospective, multicenter studies are required to confirm its predictive power, particularly for HF-related hospitalization, and to define its practical utility in clinical workflows. Collectively, this meta-analysis reinforces miRNA-21 as a potential biomarker for early detection, prognostic assessment, and personalized management of HF.

**GAMER statement:** The authors declare that no generative AI tools were used in the writing, editing, figure/table preparation, or reference management of this manuscript.

## Supplemental data

Supplemental data are available at the following link: https://www.bjbms.org/ojs/index.php/bjbms/article/view/13164/4091.

## Data Availability

The dataset used in this study is available within this manuscript and supplementary information can be requested from the corresponding author on request.
